# Gaining Momentum Around Advancing Age Inclusivity

**DOI:** 10.1093/geroni/igab046.863

**Published:** 2021-12-17

**Authors:** Carrie Andreoletti, Andrea June

**Affiliations:** 1 Central Connecticut State University, New Britain, Connecticut, United States; 2 Central Connecticut State University, Central Connecticut State University, Connecticut, United States

## Abstract

Have you already experienced some success with age friendly initiatives at your institution but are wondering how you might broaden your reach? Fostering connections across disciplines and units on your campus as well as with organizations in your community is the key to gaining momentum and advancing age inclusivity. This presentation will discuss strategies for connecting and engaging faculty, staff, students, and community members in age friendly programs and practices. We will share examples and tips for supporting others to be more age inclusive in their teaching, research, and community engagement. We will share ideas from the AFU toolkit for creating learning groups, collaborative community events, and intergenerational exchange as well as our own experience which has demonstrated that many smaller efforts over time can go a long way toward building momentum and creating a more age inclusive campus.

